# Responses in fast-spiking interneuron firing rates to parameter variations associated with degradation of perineuronal nets

**DOI:** 10.1007/s10827-023-00849-9

**Published:** 2023-04-14

**Authors:** Kine Ødegård Hanssen, Sverre Grødem, Marianne Fyhn, Torkel Hafting, Gaute T. Einevoll, Torbjørn Vefferstad Ness, Geir Halnes

**Affiliations:** 1 Department of Physics, University of Oslo, Oslo, Norway; 2 Centre for Integrative Neuroplasticity, University of Oslo, Oslo, Norway; 3 Department of Biosciences, University of Oslo, Oslo, Norway; 4 Institute of Basic Medical Sciences, University of Oslo, Oslo, Norway; 5 Department of Physics, Norwegian University of Life Sciences, Ås, Norway

**Keywords:** Perineuronal nets, Capacitance, Firing rate, PV cells, Fast-spiking interneurons, Multicompartment models of neurons

## Abstract

The perineuronal nets (PNNs) are sugar coated protein structures that encapsulate certain neurons in the brain, such as parvalbumin positive (PV) inhibitory neurons. As PNNs are theorized to act as a barrier to ion transport, they may effectively increase the membrane charge-separation distance, thereby affecting the membrane capacitance. Tewari et al. (2018) found that degradation of PNNs induced a 25%-50% increase in membrane capacitance $c_\text {m}$ and a reduction in the firing rates of PV-cells. In the current work, we explore how changes in $c_\text {m}$ affects the firing rate in a selection of computational neuron models, ranging in complexity from a single compartment Hodgkin-Huxley model to morphologically detailed PV-neuron models. In all models, an increased $c_\text {m}$ lead to reduced firing, but the experimentally reported increase in $c_\text {m}$ was not alone sufficient to explain the experimentally reported reduction in firing rate. We therefore hypothesized that PNN degradation in the experiments affected not only $c_\text {m}$, but also ionic reversal potentials and ion channel conductances. In simulations, we explored how various model parameters affected the firing rate of the model neurons, and identified which parameter variations in addition to $c_\text {m}$ that are most likely candidates for explaining the experimentally reported reduction in firing rate.

**Supplementary Information:**

The online version contains supplementary material available at 10.1007/s10827-023-00849-9.

## Introduction

The perineuronal nets (PNNs) are condensed structures of extracellular matrix that encapsulate the soma and proximal dendrites of among others parvalbumin positive (PV) inhibitory neurons in the brain (Fawcett et al., 2019). PNNs are composed of hyaluronan chains, to which chondroitin sulphated proteoglycans (CSPGs) are attached. The CSPGs in PNNs are mainly aggrecan and brevican. Both hyaluronan and chondroitin sulfate are glycosaminoglycans, which are large, unbranched, strongly negatively charged sugar molecules. The CSPGs in the PNNs are cross-linked by tenascin-R. PNNs are long-lived, stable structures hypothesized to stabilize synapses and they have to be enzymatically cleaved to allow for synapse growth (van ’t Spijker & Kwok, 2017). Furthermore, they are thought to act as a barrier to ion transport because of their massive negative charge (Morawski et al., 2015; Hanssen & Malthe-Sørenssen, 2022).

Enzymatic degradation of PNNs induces a dramatic increase in plasticity in visual cortex (Pizzorusso et al., 2002) and reduces spiking activity of putative PV neurons *in vivo* (Balmer, 2016; Lensjø et al., 2017; Christensen et al., 2021). However, the mechanisms underlying these changes remain elusive. Some experimental studies find no significant differences in the electrophysiological properties of neurons with and without PNNs, using chondroitinase ABC treatment to degrade the PNNs (Dityatev et al., 2007; Pyka et al., 2011).

**Fig. 1:**
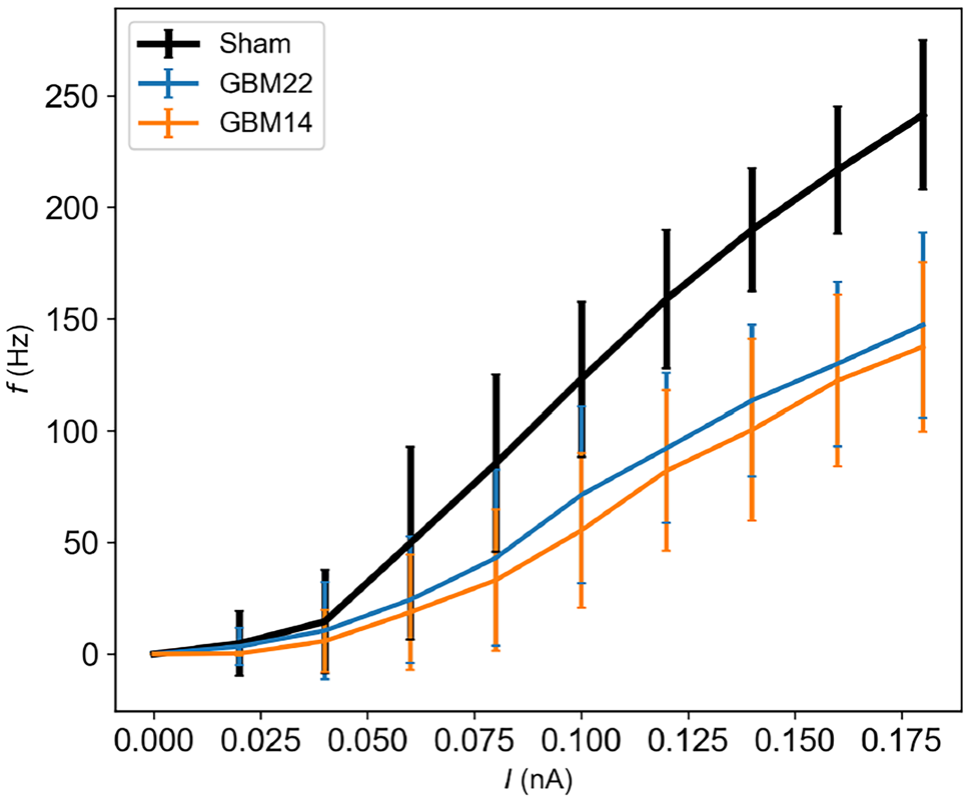
Fast-spiking interneurons without perineuronal nets show reduced firing rate in experimental data from Tewari et al. (2018). Recordings were made from brain slices from mice injected with the following: Sham - phosphate-buffered saline, GBM14 - Non-glutamate releasing tumor, GBM22 - Glutamate-releasing tumor. Measurements were made on a minimum of seven neurons for each injection type. The tumors were shown to break down PNNs in their proximity. Firing rate *f* is plotted against input current *I*. The decrease in *f* was 38% from Sham to GBM22 and 41% from Sham to GBM14, as measured for the highest input current in the figure. The data were provided by Tewari et al. (2018)

Tewari et al. (2018) performed *in vitro* measurements of capacitance and firing rate *f* under the presence of glutamate-releasing tumor GBM22 and non-glutamate releasing tumor GBM14, and found that matrix metalloproteinases (MMPs) released from the tumors disintegrated the PNNs, leading to a 25% increase in capacitance for the PNN-enwrapped interneurons and the 38% decrease in *f* seen in Fig. 1.

However, variability within their results implies that the change in capacitance in some instances could be up to 50%. As a control, they also showed that application of MMP-blocker GM6001 in the presence of tumor, resulted in normal behavior of the PNN-enwrapped neurons. In an attempt to reproduce the results *in vitro*, acute brain slices were treated with the bacterial chondroitinase-ABC (chABC) which degrades PNNs. Recording from the same neurons before and after chABC treatment showed an increased capacitance and decreased *f*. To complement their experimental findings, Tewari et al. performed simulations using a modified one-compartment version of the Hodgkin-Huxley (HH) model (as proposed by Abbott and Kepler (1990)). Similar to the experiments, the simulations showed that an increase in $C_\text {m}$ resulted in a reduced *f*. However, the effect in the model was smaller than in their experiments (Tewari et al., 2018).

Since the membrane is largely impermeable to ions, it acts as the dielectric in a capacitor which can separate a net positive charge (on one side) from a net negative charge (on the other side). PNNs have been suggested to act as an insulator that effectively acts to thicken the membrane, thereby decreasing $C_\text {m}$ by increasing the distance between the exterior and interior membrane charges Tewari et al. (2018). This provides an explanation to why PNN degradation leads to an increased $C_\text {m}$ in the experiments by Tewari et al. (2018). In principle, the insulating properties of PNNs might also lead to an increase in the membrane resistance $R_\text {m}$, but notable effects of PNN degradation on $R_\text {m}$ were not found in the experiments by Tewari et al. (2018).

Since the membrane time constant $\tau _\text {m}$ is related to $C_\text {m}$ by $\tau _\text {m}=R_\text {m}C_\text {m}$, a decrease in $C_\text {m}$ (with a fixed $R_\text {m}$) will lead to a decrease in $\tau _\text {m}$. As a decrease in $C_\text {m}$ leads to a decrease in the membrane time constant and thereby to faster membrane dynamics, we might expect a decrease in $C_\text {m}$ to increase the firing frequency *f* of the neuron. Likewise, we might expect an increase in $C_\text {m}$, e.g. due to degradation of PNNs, to reduce *f*. However, altering the time course of the membrane potential dynamics will also alter the complex interplay between various depolarizing and hyperpolarizing membrane currents through active ion channels. Hence, the relationship between $C_\text {m}$ and *f* is not trivial, and will generally depend on the ion channels that a neuron possesses, as well as its input conditions (Szlavik, 2003; Wang et al., 2012).

The one-compartment HH model used by Tewari et al. has its limitations when it comes to modeling effects of PNN changes in PV neurons. Firstly, the HH model was constructed from measurements from the squid giant axon and does not encompass the properties of mammalian PV interneurons. It is common to distinguish between two types of excitability in neurons: Type I excitability, where the neuron can fire with arbitrarily low firing frequency close to the threshold current, and Type II, where firing increases abruptly from zero to a non-zero value when the threshold is reached. The HH model has Type II excitability. Thus, the HH model does not share the dynamical properties of the PV neurons in Tewari et al.’s experiments, whose $f-I$ curves displayed a Type I excitability (Sterratt et al., 2011).

Lastly, PNNs typically enwrap only the soma and proximal dendrites of PV neurons. Using a one-compartment model, one cannot account for such geometrical specificity, as one are forced to introduce the same changes in $C_\text {m}$ over the neuron as a whole. Thus, the one-compartment HH model is not the best choice for capturing the firing characteristic of PV cells, for which there exist recent state-of-the-art multicompartment models.

In the present work, we perform a more systematic modeling study to explore possible mechanisms behind the reduction in *f* observed *in vitro* by Tewari et al. (2018). Similar results have also been observed *in vivo* (Balmer, 2016; Lensjø et al., 2017). To do this, we implement a range of models taken from the literature, including models constrained to electrophysiological data from PV cells. We find that in none of the models, the moderate capacitance changes observed in the experiments of Tewari and co-workers are sufficient to explain the measured changes in *f*, suggesting that PNN degradation also affects other cellular properties. We therefore expand the study, suggesting additional candidate mechanisms that may have contributed to the experimentally observed changes in *f*.

## Methods

In the present study we try to explain the experiments by Tewari et al. (Fig. 1) in terms of changes in specific membrane capacitance $c_\text {m}$ (capacitance per membrane area), conductance values $\bar{g}_X$ for maximally open ion channels, and reversal potentials $E_X$ for various ion channels *X*. The effects of these candidate mechanisms on the firing frequency *f* were studied in nine models, as presented in Table 1: a one-compartment HH model (OC), a ball-and-stick HH model (BAS) and three models developed by the Allen Institute for Brain science (A1, A2, A3). The multi-compartmental models come in two versions: one where $c_\text {m}$ is varied everywhere (all), and one where $c_\text {m}$ is varied only on the soma and proximal dendrites (sprx), as the PNNs are normally believed to encapsulate these parts of the neuron (Sorg et al., 2016). The proximal part of the dendrites was set to encompass every segment of the dendrite less than 3.5 soma lengths away from the cell body as measured by path distance along the neurites.

**Table 1:** Models used in this study. OC is a one-compartment Hodgkin-Huxley model, BAS is a ball-and-stick model with passive dendrite and Hodgkin-Huxley mechanisms in the soma, and A1-A3 are three PV interneuron models developed by the Allen Institute for Brain Science constrained to morphological and electrophysiological data from real PV neurons. $c_{\text {m,all}}$ - $c_\text {m}$ is changed everywhere, $c_{\text {m,sprx}}$ - $c_\text {m}$ is changed only on the soma and proximal dendrites, Multicomp. - Multicompartment

Model	Multicomp.	HH	Allen	$c_{\text {m,all}}$	$c_{\text {m,sprx}}$
OC		$\checkmark$		$\checkmark$	
BAS, all	$\checkmark$	$\checkmark$		$\checkmark$	
BAS, sprx	$\checkmark$	$\checkmark$			$\checkmark$
A1, all	$\checkmark$		$\checkmark$	$\checkmark$	
A2, all	$\checkmark$		$\checkmark$	$\checkmark$	
A3, all	$\checkmark$		$\checkmark$	$\checkmark$	
A1, sprx	$\checkmark$		$\checkmark$		$\checkmark$
A2, sprx	$\checkmark$		$\checkmark$		$\checkmark$
A3, sprx	$\checkmark$		$\checkmark$		$\checkmark$

All models were based on a Hodgkin-Huxley type formalism, where the membrane potential dynamics in a given compartment *j* (with membrane area $A_j$) is governed by the differential equation,

1 $$c_{\text {m},\kern0.1500emj}\frac{dV_{\textrm{m},\kern0.1500emj}}{dt} = -i_{\textrm{L},\kern0.1500emj} - \sum _X{i_{X,\kern0.1500emj}} +\frac{I_{\text {stim},\kern0.1500emj}}{A_j} + \frac{I_{j-1,\kern0.1500emj}-I_{j,\kern0.1500emj+1}}{A_j}.$$

Here, $I_{j-1,j}$ and $I_{j,j+1}$ represent incoming and outgoing axial currents, respectively, from neighboring compartments (relevant only for multicompartment models). Parameters affecting the axial currents explicitly were not changed in this project. The remaining currents are membrane current densities for the leakage channel

2 $$\begin{aligned} i_\text {L} = \bar{g}_L(V-E_\text {L}), \end{aligned}$$

and various ion channels

3 $$\begin{aligned} i_X = \bar{g}_X m^\alpha h^{\beta }(V-E_X), \end{aligned}$$

the assembly of which differed between the different models. In this general formalism, *m* and *h* are so-called gating variables, opening and closing the ion channels as a function of membrane potential and time, while $\alpha$ and $\beta$ represent the number of gates of each type. Whereas these gating variables express genetically coded kinetics of the ion channel, $\bar{g}_X$ represents the conductance when all channels of type *X* are fully open. $E_X$ is the reversal potential for the ion channel of type *X*.

The Hodgkin-Huxley model contains conductances for sodium, potassium and a leak conductance. The Allen models incorporate six different potassium conductances, a voltage dependent-sodium conductance, two calcium conductances and a general cation conductance, along with a passive leak conductance. The calcium reversal potential $E_\text {Ca}$ can change dynamically, as a function of intracellular calcium dynamics, which was explicitly accounted for in the Allen models.

### Implementation

Simulations with varying input currents, conductances, reversal potentials and specific capacitances were run in NEURON (Carnevale & Hines, 2006) with LFPy (Hagen et al., 2018) as a wrapper. Time and somatic membrane potential was written to file and analyzed using custom-made scripts. The time step of all simulations was set to dt$=0.0078125$ ms, and all simulations were run for 600 ms before the recording started.

### One-compartment model

The one-compartment model was simulated with the use of NEURON’s built-in Hodgkin-Huxley mechanisms. The length and diameter of the one-compartment model was set to 10 $\mu$m.

### Ball-and-stick models

The ball-and-stick models were constructed by connecting a soma compartment to a dendrite compartment in NEURON. The dendrite was divided into segments of 5 $\mu$m for the simulations. The diameter of the soma was 10 $\mu$m.

The dendrite length was set to $l=1000$ $\mu$m and the dendrite diameter to $d=1$ $\mu$m. NEURON’s HH mechanisms were inserted into the soma and passive mechanisms were inserted into the dendrite. The leak potential of the dendrite was set to -65 mV to match the resting potential of the soma, and the leak conductance was set to 0.0003 S/cm$^2$ in the dendrite. The axial resistivity $R_\text {a}$ was set to 100 $\Omega$cm.

### Allen models

Mechanisms and morphologies of PV-neurons were taken from the Allen Brain Atlas’ Cell Database (Allen Institute for Brain Science, 2022) and run in NEURON. Allen model 1, 2 and 3 are the perisomatic models (meaning that active conductances were only included in the soma compartment) of cells with CellID 471077857, 487667205 and 396608557, respectively. The Allen group removed all axon compartments and replaced them with an axon initial segment of 60 $\mu$m length and 1 $\mu$m diameter before performing the fit. For consistency, we used the same axon, giving it two compartments of two segments each.

The Allen models included the following mechanisms (Allen Institute for Brain Science, 2017):

- Hyperpolarization-activated cation conductance $g_\text {h}$
- Markov-style formulation $\text {Na}^+$ channel conductance $g_\text {NaV}$
- $$\text {K}_{\text {v}}\text {1-like}$$ $$\text {K}^+$$ conductance $$g_\text {Kd}$$
- Kv2-like conductance $g_\text {Kv2like}$
- Fast-inactivating ($\text {K}_{\text {v}}\text {4-like}$) K$^+$ conductance $g_\text {KT}$
- Kv3-like conductance $g_\text {Kv3}$
- M-type K$$^+$$ conductance $$g_\text {Imv2}$$
- SK-type Ca$^{2+}$-activated K$^+$ conductance $g_\text {SK}$
- High-voltage-activated Ca$^{2+}$ conductance $g_\text {CaHVA}$
- Low-voltage-activated Ca$^{2+}$ conductance $g_\text {CaLVA}$
- A passive conductance $$g_\text {L}$$

The conductances were fit to experimental recordings by the Allen Institute for Brain Science and differed from model to model.

#### Changes in Nernst potentials

The reversal potential $E_k$ of ion species *k* is

4 $$\begin{aligned} E_k=\frac{RT}{z_kF}\ln {\frac{\left[ k\right] _\text {out}}{\left[ k\right] _\text {in}}}, \end{aligned}$$

where *R* is the gas constant, *F* is Faraday’s constant, $z_k$ is the valency and $\left[ k\right] _\text {in}$ and $\left[ k\right] _\text {out}$ are the intracellular and extracellular concentrations. In the Allen models, $E_{\text {Ca}}$ varied dynamically through equations for calcium dynamics proposed by Destexhe et al. (1994) and Eq. (4), as Ca$^{2+}$ currents affected the intracellular Ca$^{2+}$ concentration. When we investigated hypothesized effects on $E_{\text {Ca}}$ due to PNN degradation, we changed $E_{\text {Ca}}(t=0)$ by specifying fixed changes in the extracellular Ca$^{2+}$ concentration, which did not vary dynamically in the model.

### Simulation protocol

Frequency-input ($f-I$) curves were computed by injecting constant currents of different amplitude into the soma. The current was increased in increments of 0.01 nA up to the value where the neuron was driven into depolarization block and was no longer able to fire action potentials. The input current duration was set to 1000 ms for the HH models. The Allen model neurons often exhibited late-onset spiking for current injections close to the threshold current. They were therefore stimulated by currents of 2000 ms duration, with the spiking frequency obtained from the last 1000 ms of the stimulus. The same protocol was used to find the threshold current, with a resolution of 0.001 nA. Spike frequencies and thresholds were found for sustained firing: Spikes were only counted if at least one spike occurred in the latter half of the stimulation interval, that is the last 1000 ms for the Allen models and the last 500 ms for the HH models. A spike was detected if the voltage at one point in time was larger than for both the preceding and the following time step, while also being larger than -20 mV.

## Results

We studied how $f-I$ curves in the nine models described in the methods section were sensitive to a selection of model parameters, including the specific membrane capacitance $c_\text {m}$, maximal conductances $\bar{g}_X$ for various ion channels, and ionic reversal potentials $E_k$.

### Effects of $c_\text {m}$ on firing properties

**Fig. 2:**
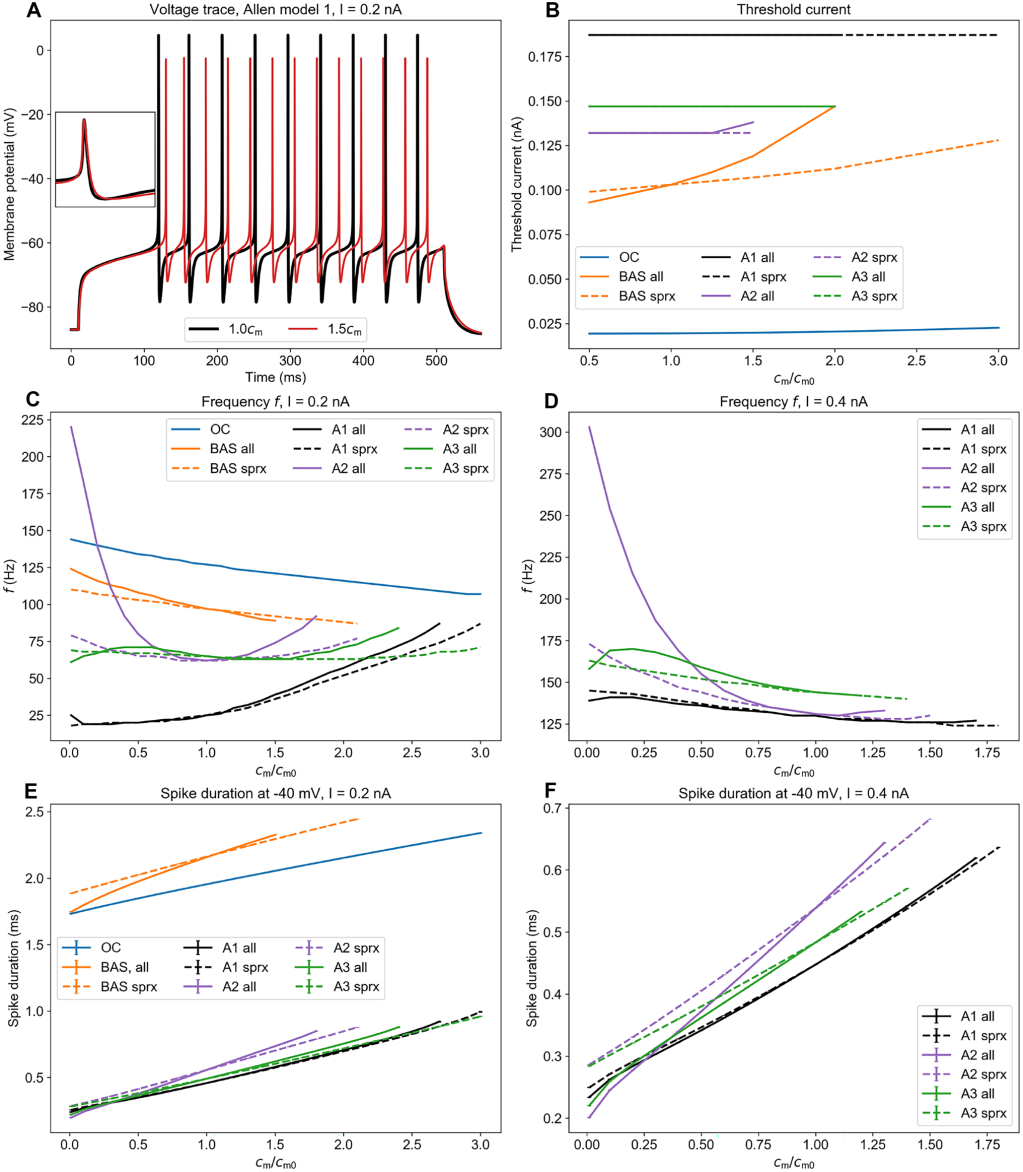
Properties of the neuron model as a function of specific membrane capacitance $c_\text {m}$. $c_\text {m}0$ is the default value of $c_\text {m}$ in the model. **A** Voltage trace for Allen model 1 for default $c_\text {m}$ and $1.5c_\text {m}$. Inset: normalized voltage trace over the duration of one peak. The two traces have been shifted to align the peak maxima, **B** Threshold current vs $c_\text {m}$, **C** Frequency *f* vs $c_\text {m}$ for all models for input current I = 0.2 nA, **D** *f* vs $c_\text {m}$ for the Allen models for I = 0.4 nA, **E** Spike duration (defined as the width of the spike at -40 mV) for I = 0.2 nA, **F** Spike duration at -40 mV for I = 0.4 nA. Note that the one-compartment model and the ball-and-stick model do not fire for $I=0.4$ nA. OC - one-compartment model, BAS - ball-and-stick model, A1 - Allen model 1, A2 - Allen model 2, A3 - Allen model 3, all - $c_\text {m}$ changed at every segment of the neuron, sprx - $c_\text {m}$ only changed at the soma and proximal dendrites

The firing properties of all models were sensitive to the value of $c_\text {m}/c_\text {m0}$, where $c_\text {m0}$ is the model’s default value of the capacitance. An example illustration is given in Fig. 2A showing the voltage trace of Allen model 1, where an increasing $c_\text {m}$ lead to a broadening of the spikes, a lower spike amplitude and a decreased firing rate. The broadening was general for all models (Fig. 2E, F), as expected, since $c_\text {m} \propto \tau _\text {m}$ should slow down the membrane dynamics.

Note that the Allen models exhibit narrower spikes than the HH models, resembling the short spike duration observed in PV cells (Bartos & Elgueta, 2012).

The effect of $c_\text {m}$ on the threshold current for firing onset is shown in Fig. 2B. The threshold current remained constant or varied only slightly with the capacitance, except for the ball-and-stick model. However, in none of the models the onset was shifted when varying $c_\text {m}$ over the interval relevant under Tewari et al.’s experiments (i.e. from default to a factor 1.5 increase). This held both for simulations where $c_\text {m}$ had only been altered in the soma and proximal dendrites, as indicated by dashed lines, and simulations where $c_\text {m}$ was changed everywhere, as indicated by solid lines.

There was no clear general trend shared among the models in terms of how the firing frequency *f* depended on $c_\text {m}$. Over the same $c_\text {m}$ interval, *f* increased with $c_\text {m}$ in some models, while it decreased with $c_\text {m}$ in others (Fig. 2C, D). This was also the case for the $c_\text {m}$ interval relevant under the experiments by Tewari et al. However, for the strongest of the current injections considered (0.4 nA in Fig. 2D), all models except Allen model 2 (which stopped firing at $\sim$ 25% increase in $c_\text {m}$) showed a decreasing trend in *f* with $c_\text {m}$. This suggests that at least the maximal firing rate in these models should be reduced, like in the experiments, when $c_\text {m}$ is increased (as an effect of PNN degradation).

For all the Allen models, *f* vs $c_\text {m}$ varied less when $c_\text {m}$ was only changed in the soma and proximal dendrites, which is to be expected as we altered $c_\text {m}$ on a smaller part of the neuron. For Allen model 3, for instance, this graph appeared far less curved when $c_\text {m}$ was changed in the soma and the proximal dendrites compared to when $c_\text {m}$ was changed everywhere. However, within the range $c_\text {m}/c_\text {m0}\in [1.0,1.5]$, the difference between the two cases was relatively small. In the following, we therefore show results only for the supposedly more realistic case where PNN degradation is assumed to alter $c_\text {m}$ only on the soma and proximal dendrites (results for $c_\text {m}$ altered everywhere is found in Supplementary Fig. 1).

**Fig. 3:**
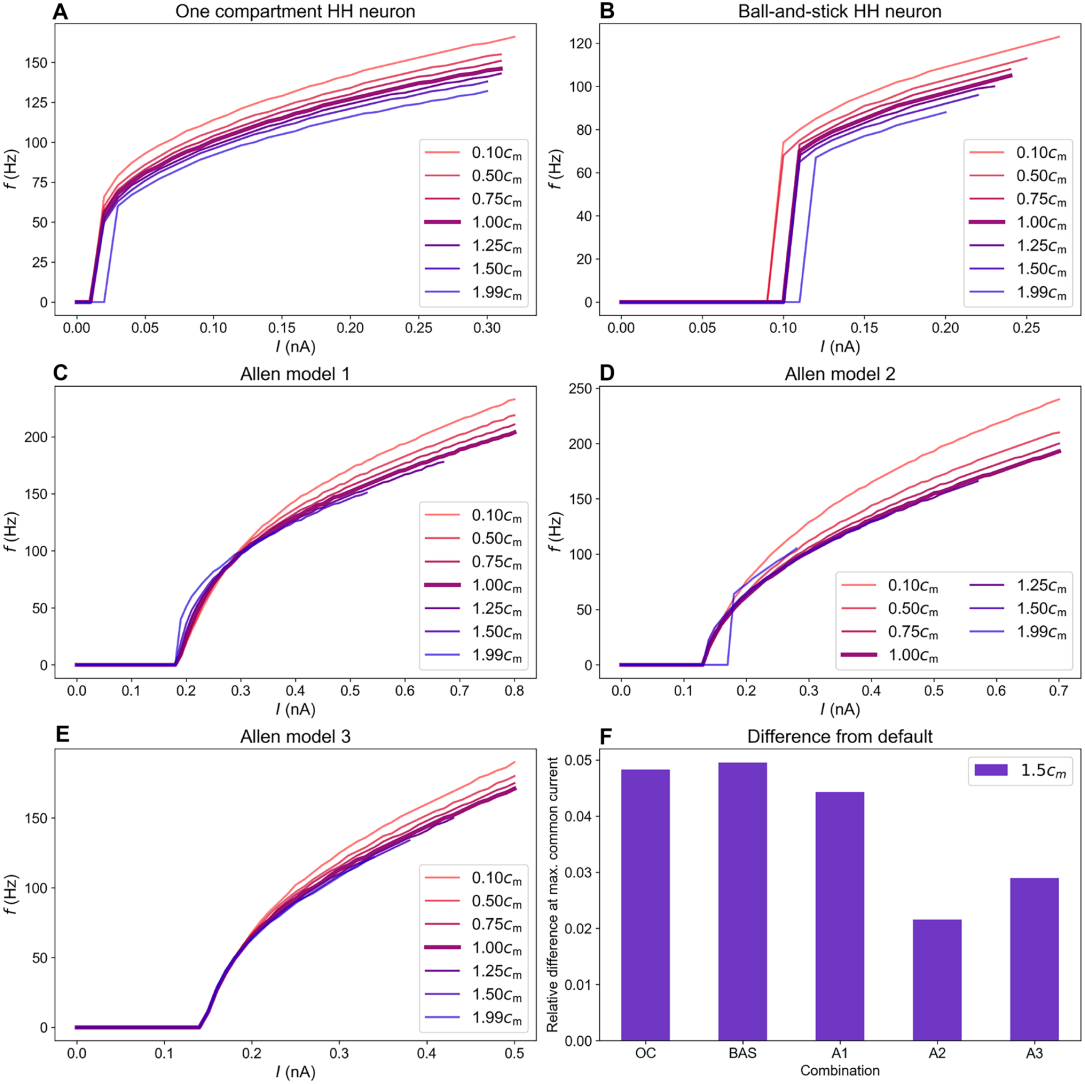
Frequency-input curves for selected values of $c_\text {m}$ for the various models. $c_\text {m}$ is altered in the soma and proximal dendrites. **A** The one-compartment Hodgkin-Huxley model, **B** The ball-and-stick Hodgkin-Huxley model, **C** Allen model 1, **D** Allen model 2, **E** Allen model 3, **F** The relative difference in *f* between the $1.0c_\text {m}$- and $1.5c_\text {m}$ curves computed at the largest current that gave sustained firing in both cases

To compare with the $f-I$ curves of Tewari et al. (2018) (Fig. 1) we stimulated the different neuron models with a range of input currents for various values of $c_\text {m}$. Except for stimuli near the onset threshold, all models displayed a reduction in *f* when increasing $c_\text {m}$ (Fig. 3).

In addition to affecting the firing rate, changes in $c_\text {m}$ caused a shift in the spiking onset threshold in some of the models (Fig. 3). However, in none of the models the onset was shifted when varying $c_\text {m}$ over the interval relevant under the Tewari et al.’s experiments.

The HH models (Fig. 3A, B) exhibited type II firing, meaning that the firing rate increases abruptly from zero to a higher value when the threshold current is reached. However, Tewari et al. observed Type I firing in their experiments. The HH models are therefore not ideal for simulating PV cells.

The Allen models, which were constructed based on morphological and electrophysiological recordings from real PV cells, had $f-I$ curves that were more similar to the the experimental recordings. For Allen model 1 the firing frequency increased with $c_\text {m}$ for input currents close to the threshold current (Fig. 3C). At an input current of around $I=0.28$ nA, the $f-I$ curves crossed, after which the firing frequency decreased with $c_\text {m}$. For Allen model 2, the $f-I$ curve crossings started closer to the threshold current and were less pronounced (Fig. 3D). For a relatively larger range of input currents *f* decreased with increasing $c_\text {m}$. For Allen model 3, the firing frequency was approximately equal for all $c_\text {m}$ for stimuli up to $I=0.18$ nA, after which *f* started to decrease as $c_\text {m}$ was increased (Fig. 3E).

None of the $f-I$ curves in Fig. 3 show a sufficient reduction in firing when reducing $c_\text {m}$ by 25 or 50% to explain the observations in the experiments by Tewari et al. (as seen from Fig. 3G). In other words, changing $c_\text {m}$ was on its own not enough to reproduce their findings. We therefore hypothesized that PNN degradation affected additional mechanisms which also contributed to the observed reduction in *f*. It has been reported that PNNs might affect both local concentrations of ions (Morawski et al., 2015; Burket et al., 2021) or currents through ion channels (Vigetti et al., 2008; van ’t Spijker & Kwok, 2017). In the following sections, we have therefore explored how variations in reversal potentials $E_k$ and ion channel conductances $\bar{g}_X$ affect the firing frequency of our model neurons.

As the HH models contained relatively few of the membrane mechanisms present in PV neurons, and also produced type II firing unlike the type I firing seen in Tewari et al.’s experiments, we excluded them from our further analyses, and focused on the Allen models.

### Effects of reversal potentials on firing rates

**Fig. 4:**
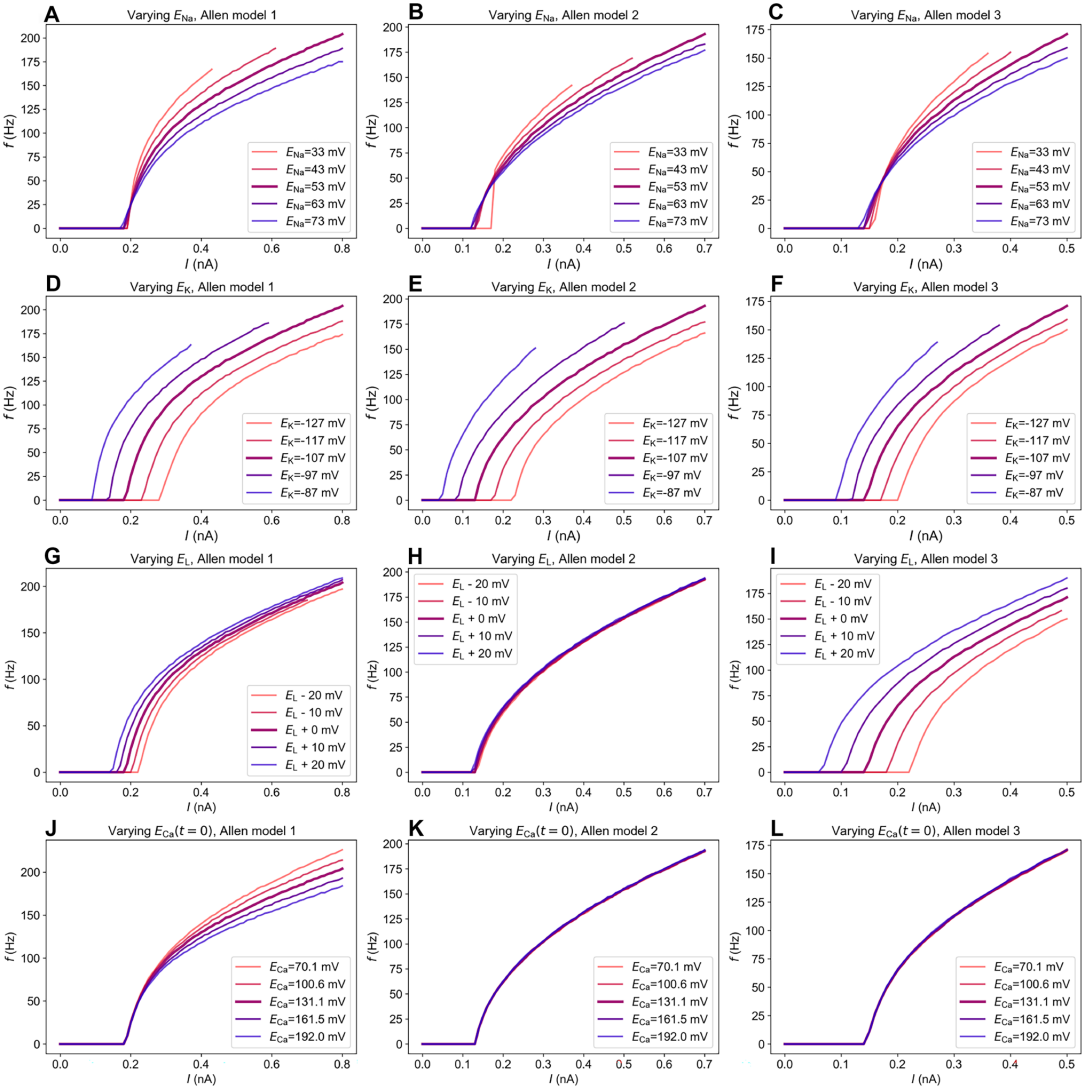
Frequency-input curves when varying the different reversal potentials in the Allen models. Note that the reversal potential of calcium in the Allen models was found using calcium dynamics together with Eq. (4), so $E_{\text {Ca}}$ is given at $t=0$ ms and will vary throughout the simulations

PNNs have been shown to be involved in the regulation of ionic concentrations (Morawski et al., 2015; Burket et al., 2017), and it is therefore likely that PNN degradation will lead to changes in ionic reversal potentials. This may in turn have dramatic consequences for neural firing properties, as has been the topic of many previous studies (Kager et al., 2000; Wei et al., 2014; Sætra et al., 2020). In order to gauge their general effect on the firing in PV neurons, all reversal potentials in the Allen models were changed separately by up to $\pm 20$ mV, as shown in Fig. 4.

The simulations suggested that, among the reversal potentials, $E_\text {Na}$ (all Allen models: Fig. 4A-C) and $E_\text {Ca}$ (in Allen model 1: Fig. 4J) seemed the most likely candidates to have contributed during the experiments by Tewari et al. Both these led to moderate changes in the firing rate without strongly affecting the onset of firing. In contrast, changes in $E_\text {K}$ and $E_\text {L}$ (Fig. 4D-I) caused large shifts in firing onset not seen in the experiments, or no effect at all (Fig. 4H).

### Effect of conductances on firing rates

**Fig. 5:**
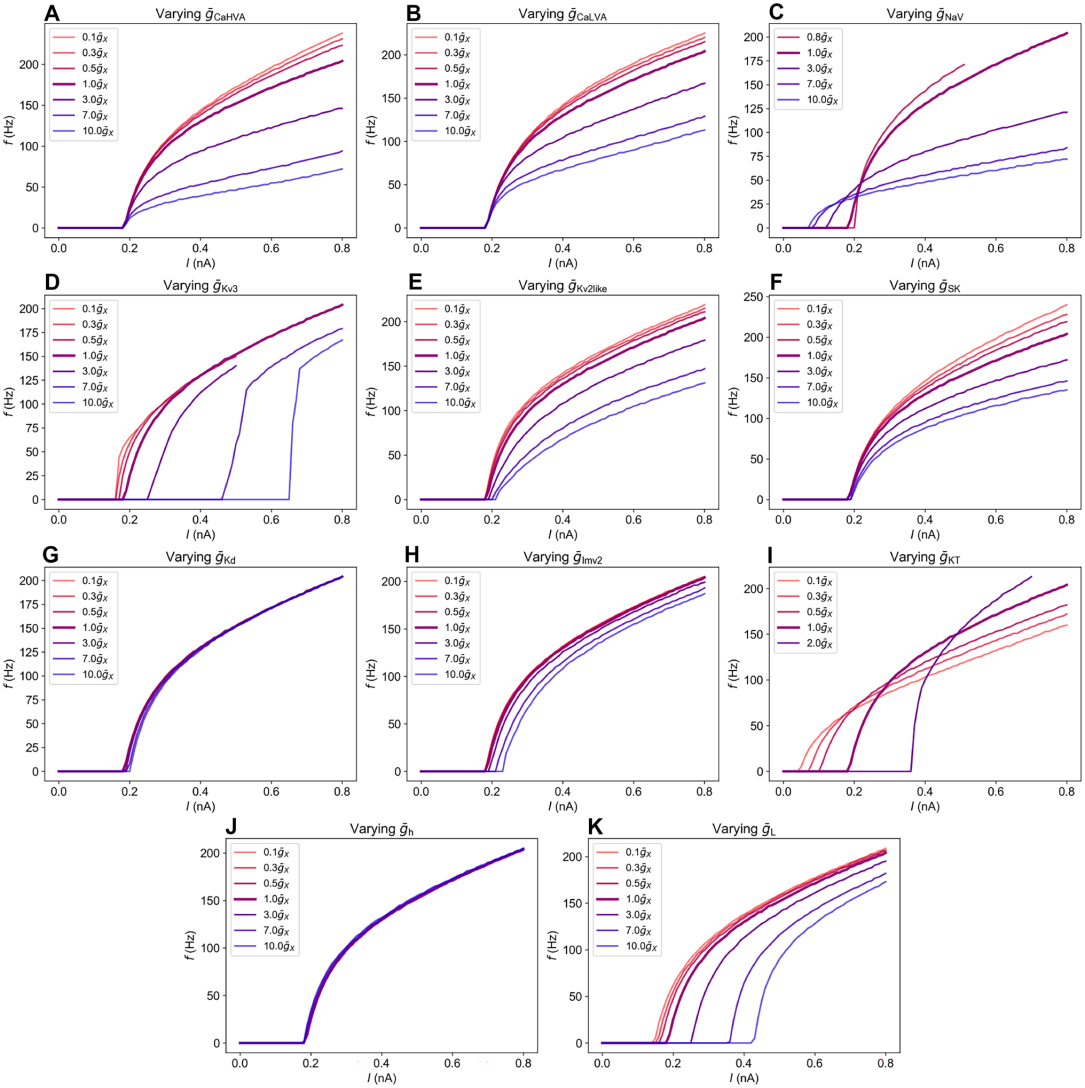
Frequency-input curves when varying different conductances in Allen model 1. $\bar{g}_X$ is the default value of the conductance. **A** $\bar{g}_{\text {CaHVA}}$, **B** $\bar{g}_{\text {CaLVA}}$, **C** $\bar{g}_{\text {NaV}}$, **D** $\bar{g}_{\text {Kv3}}$, **E** $\bar{g}_{\text {Kv2like}}$, **F** $\bar{g}_{\text {SK}}$, **G** $\bar{g}_{\text {Kd}}$, **H** $\bar{g}_{\text {Imv2}}$, **I** $\bar{g}_{\text {KT}}$, **J** $\bar{g}_{\text {h}}$, **K** $\bar{g}_{\text {L}}$

To gauge the effect of conductance changes on $f-I$ curves, we varied all conductances one-by-one over an interval ranging from 0.3$\bar{g}_X$ to 10.0$\bar{g}_X$, where $\bar{g}_X$ is the default maximal conductance (for fully open ion channels). Among the nine models, Allen model 1 responded most strongly to conductance changes. We therefore show results only for that model (Figure 5). The effect of conductance on Allen models 2 and 3 is shown in (Supplementary Figs. 2 and 3).

#### Calcium conductances

In many neuron types, inward depolarizing Ca$^{2+}$ currents trigger outward hyperpolarizing K$^{+}$ currents through Ca$^{2+}$-activated K$^{+}$ channels (see e.g. Destexhe & Sejnowski (2003) or Halnes et al. (2011)). Hence, whether the overall effect of a Ca$^{2+}$ current leads to an increased or decreased firing rate generally depends on the neuron’s ion channel composition.

In Allen model 1, the direct depolarizing effect associated with inward Ca$^{2+}$ currents was much smaller than the secondary hyperpolarizing effects associated with the activation of Ca$^{2+}$- activated SK channels. Increasing $\bar{g}_{\text {CaHVA}}$ thus had a negative effect on the firing rate in this model (Fig. 5A). Increasing $\bar{g}_{\text {CaHVA}}$ by factors 3, 7 and 10, lead to quite pronounced decreases in *f* by 28%, 54% and 65%, respectively, at the maximal current injection considered (0.8 pA). In comparison, the decrease in *f* (at the maximal current injection) in Tewari et al.’s experiments was 38%. The increased conductance did not lead to a shift in the onset of firing. Likewise, reductions in *f* (without a shift in the onset threshold) could also be obtained by an increase in $\bar{g}_{\text {CaLVA}}$ (Fig. 5B).

Combined with changes in $c_\text {m}$ and possibly other mechanisms, $\bar{g}_{\text {CaHVA}}$ and $\bar{g}_{\text {CaLVA}}$ could be candidate mechanisms for explaining effects of PNN degradation on firing properties. However, we did not find experimental studies in support of the notion that PNN degradation should increase Ca$^{2+}$ conductances. Contrarily, in retinal photoreceptors, chondroitin sulfates, which are key components of the PNNs, were found to shift the activation curve of unspecified calcium channels towards lower voltages (Vigetti et al., 2008). Hence, if removing PNNs means removing chondroitin sulfates, we would expect activation to shift towards higher values, resulting in generally reduced calcium current $I_{\text {Ca}}$. Likewise, in experiments on hippocampal slices, (Kochlamazashvili et al., 2010) found that $I_{\text {CaHVA}}$ was reduced upon breakdown of PNN component hyaluronan by hyaluronidase, and increased when hyaluronan was added to the hyaluronidase-treated neurons. If anything, the cited experiments thus suggest that PNN degradation should decrease overall calcium currents, rather than increase them, as we needed to do to reduce *f* in Allen model 1. Hence, we do no not consider $\bar{g}_{\text {CaLVA}}$ or $\bar{g}_{\text {CaHVA}}$ as main candidates for explaining Tewari et al.’s results.

We note that while $\bar{g}_{\text {CaHVA}}$ had almost no effect on *f* in Allen models 2 and 3 (Supplementary Figs. 2A and 3A), increases in $\bar{g}_{\text {CaLVA}}$ had a small positive effect on *f* in these models (Supplementary Figs. 2B and 3B). The latter suggests that in these models, the depolarizing effect of $I_{\text {CaLVA}}$ dominated over indirect hyperpolarizing effects via SK activation. However, *f* was insensitive to reductions in $\bar{g}_{\text {CaHVA}}$ and $\bar{g}_{\text {CaLVA}}$ in these models. Hence, the decrease in *f* observed in Tewari et al.’s experiments could not be obtained by reducing Ca$^{2+}$ conductances in any of the Allen models.

#### Sodium conductance

An increase in $\bar{g}_{\text {NaV}}$ lead to a downward (towards lower input) shift in the onset of firing (Fig. 5C) in Allen model 1, and thus and increased *f* for weak stimuli. However, the $f-I$ curves for various $\bar{g}_{\text {NaV}}$ crossed at about $I=0.22$ nA, and for input stronger than this, increase in $\bar{g}_{\text {NaV}}$ caused a decrease in *f*, as has been seen in a previous modeling study (Kispersky et al., 2012).

There is experimental support that PNNs affect NaV currents. Tenascin-C and net component tenascin-R have been found to play a crucial role in localizing NaV channels in the axon initial segment and nodes of Ranvier (Srinivasan et al., 1998), and tenascin-R has also been found to increase the maximum amplitude of NaV currents when in solution, thus indicating an increase in $\bar{g}_{\text {NaV}}$ (Xiao et al., 1999).

As tenascin-R is a crosslinker in the nets, it is unclear whether it would get close enough to the NaV channels to affect them when present in intact PNNs. It is possible that removing the nets would lead to free tenascin-R and hence increased $\bar{g}_{\text {NaV}}$, but this effect might be transient due to diffusion of tenascin-R away from the cell surface. If tenascin-R lingers near the cell membrane after dissolving PNNs, a resulting increase in $\bar{g}_{\text {NaV}}$ could, as we saw in Fig. 5C, partially explain the decrease in firing in Fig. 1. However, increases in $\bar{g}_{\text {NaV}}$ produced pronounced shifts in the onset of firing not seen in the Tewari et al.’s experiments, and changes in $\bar{g}_{\text {NaV}}$ thus does not seem like a main candidate for explaining the experiments.

#### Potassium conductance: $\bar{g}_{\text {Kv3}}$

Ion channel Kv3.1b is often highly expressed in PV neurons, which are often enwrapped in PNNs (Favuzzi et al., 2017). Experiments have also suggested that PNNs affect Kv3.1b channels. In brevican knock-out mice, clustering of these channels were altered, and active Kv3.1b was increased (Favuzzi et al., 2017). As PNNs contain brevican, it thus seems natural to expect that PNN degradation should lead to an increase in Kv3.1b conductance and hence $\bar{g}_{\text {Kv3}}$.

The above evidence suggests that effects of PNN on $\bar{g}_{\text {Kv3}}$ could be an important contributor to the reduction in *f* seen in Fig. 1. However, increasing $\bar{g}_{\text {Kv3}}$ only gave a small reduction in *f*, but a pronounced shift towards higher input in the onset of firing (Fig. 5D), not seen in Tewari et al.’s experiments. According to the simulations, $\bar{g}_{\text {Kv3}}$ is thus not a good candidate mechanism for explaining Tewari et al.’s experiments.

#### Potassium conductance: $\bar{g}_{\text {SK}}$ and $\bar{g}_{\text {Kv2like}}$

Moderate and quite similar reductions in *f* could be obtained by increasing $\bar{g}_{\text {Kv2like}}$ (Fig. 5E) and $\bar{g}_{\text {SK}}$ (Fig. 5F). Neither of these mechanisms affected the onset of the $f-I$ curve significantly. The increase in $\bar{g}_{\text {SK}}$ has experimental support, as attenuation of the extracellular matrix through application of chondroitinase ABC have been shown to upregulate SK-channels in hippocampal neurons, leading to an increase in $I_{\text {SK}}$ by, on average, a factor 3 (see Fig. 2f in Dembitskaya et al. (2021)). When it comes to $\bar{g}_{\text {Kv2like}}$, we found no mention in the literature as to whether it is affected by PNNs. As the curves look promising and the literature does not exclude them, we consider both these conductances as candidate mechanisms for explaining parts of the reduction in *f* found in the experiments of Tewari et al.

#### Other potassium conductances

The K$^+$ conductances $\bar{g}_{\text {Kd}}$ (Fig. 5G) and $\bar{g}_{\text {Imv2}}$ (Fig. 5H) had little impact on *f*. Also, we have not found any mentions in the experimental literature suggesting that PNN affect these currents, and do not consider them as candidates for explaining Tewari et al.’s experiments.

In contrast, $\bar{g}_{\text {KT}}$ induced a clear shift in the onset of firing, as seen from Fig. 5I. Its $f-I$ curves (for various values of $\bar{g}_{\text {KT}}$) crossed at different input currents. For low input currents, *f* decreased with increasing $\bar{g}_{\text {KT}}$, while for larger input currents *f* increased with decreasing $\bar{g}_{\text {KT}}$. Due to the relatively large shifts and lack of mention in the literature, we do not consider $\bar{g}_{\text {KT}}$ as a main candidate for explaining Tewari et al.’s experiments.

#### $\bar{g}_{\text {h}}$

The hyperpolarization activated $I_\text {h}$ current was almost inactive during the depolarizing current injections used in our simulations, and presumably also in Tewari et al.’s experiments. Changing $\bar{g}_{\text {h}}$ in Allen model 1 thus had almost no impact on its $f-I$ curves (Fig. 5J). Due to its low impact on the firing frequency, we conclude that $\bar{g}_{\text {h}}$ is not a candidate mechanism for explaining the reduction in *f* found in Fig. 1. We note that chondroitin sulfates, which are present in PNNs have been found to shift the activation curve of $I_\text {h}$ in photoreceptors (Vigetti et al., 2008), but PNNs were not found to have any effect on $I_\text {h}$ in deep cerebellar nuclei (Hirono et al., 2018). As previously explained, we have focused on conductances of various channels, and have not tried to account for activation kinetics.

#### Leak conductance

A decrease in *f* could also be obtained by increasing the leak conductance $\bar{g}_{\text {L}}$ (Fig. 5K). However, similarly to $\bar{g}_{\text {KT}}$, $\bar{g}_{\text {L}}$ induced a clear shift in the onset of firing. Also, changes in the membrane resistance consistent with a change in $\bar{g}_{\text {L}}$ were not found in the experiments by Tewari et al. (2018). We therefore do not consider $\bar{g}_{\text {L}}$ as a main candidate for explaining Fig. 1.

### A combinatorial explanation

In the experiments by Tewari et al. (2018), PNN degradation lead to a maximum reduction in $c_\text {m}$ by 50%. As the simulations in Fig. 3G suggested, such a change in $c_\text {m}$ did reduce the firing rate in fast-spiking interneuron, but not sufficiently to explain the experiments in Fig. 3G). The parameter explorations in Sections 3.2 and 3.3 allowed us to identify possible candidate mechanisms that, combined with the observed change in $c_\text {m}$, could explain the drop in *f* found in the experiments.

According to the simulations, $\bar{g}_\text {Kd}$, $\bar{g}_\text {Imv2}$ and $\bar{g}_\text {h}$ are unlikely candidates since they had close to no effect on the $f-I$ curve. The conductances $\bar{g}_\text {L}$, $\bar{g}_\text {Kv3}$ and $\bar{g}_\text {NaV}$ and the reversal potentials $E_\text {K}$ and $E_\text {L}$ are unlikely candidates since varying them introduced large shifts in the onset of the $f-I$ curves not observed by Tewari et al. $f-I$ curves resembling those in Fig. 1 could be obtained by upregulating the Ca$^{2+}$ conductances $\bar{g}_\text {HVA}$ and $\bar{g}_\text {LVA}$. However, such upregulations are in conflict with previous experimental studies suggesting that PNN degradation should rather lead to a down-regulation of the mechanisms in question. Ruling out the above parameters, we are left with four possible candidate mechanisms: the reversal potentials $E_{\text {Na}}$ and $E_{\text {Ca}}$, and the conductances $\bar{g}_\text {SK}$ and $\bar{g}_\text {Kv2like}$.

Upregulating the conductances $\bar{g}_\text {SK}$ and $\bar{g}_\text {Kv2like}$, both present in the Allen PV cell models, had an effect on the $f-I$ curve similar to those seen in Fig. 1. Among these, upregulation of $\bar{g}_\text {SK}$ by PNN degradation is supported by previous experiments, while we found no mention in the literature of PNN effects on $\bar{g}_\text {Kv2like}$. Likewise, increasing $E_{\text {Na}}$ and $E_{\text {Ca}}$ also lead to the desired reduction in *f*. PNNs have been shown to accumulate cationic molecules and may provide ion sorting on neuronal membranes (Morawski et al., 2015; Burket et al., 2017). The notion that PNN degradation should affect ionic reversals reversal potentials is thus not unlikely.

**Fig. 6:**
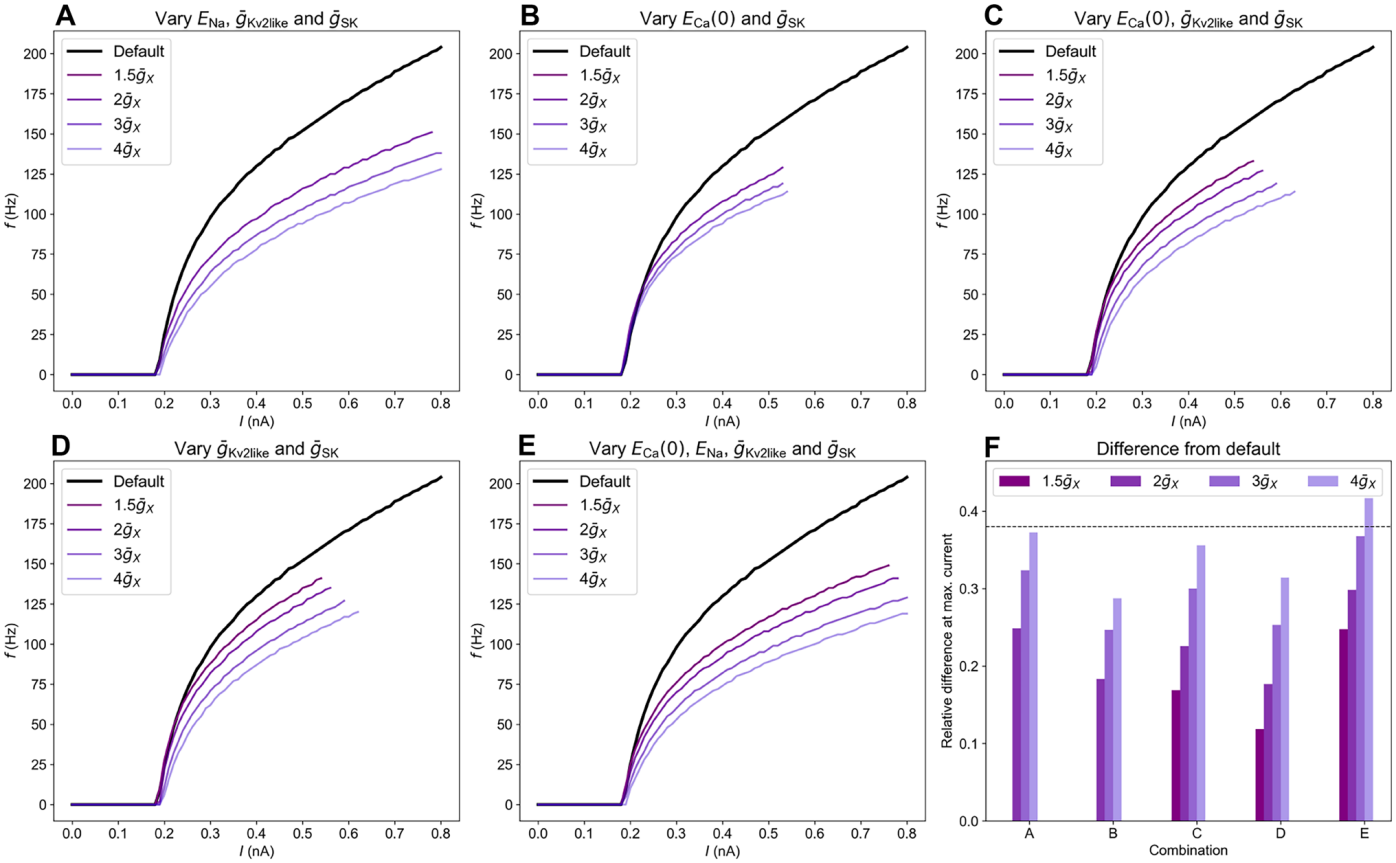
Frequency-input curves of Allen model 1 when varying $c_\text {m}$ and **A** $E_{\text {Na}}$, $\bar{g}_{\text {Kv2like}}$ and $\bar{g}_{\text {SK}}$, **B** $E_{\text {Ca}}(t=0)$ and $\bar{g}_\text {SK}$, **C** $E_{\text {Ca}}(t=0)$, $\bar{g}_\text {Kv2like}$ and $\bar{g}_\text {SK}$, **D** $\bar{g}_\text {Kv2like}$ and $\bar{g}_\text {SK}$, **E** $E_{\text {Ca}}(t=0)$, $E_{\text {Na}}$, $\bar{g}_\text {Kv2like}$ and $\bar{g}_\text {SK}$, **F** Relative difference between each parameter combination and default at the largest current that gave sustained firing in both cases. The horizontal dashed line indicate the relative difference between *f* of Sham and GBM22 in Tewari et al.’s experiments. The difference between Sham and GBM14 is a bit larger. Default - default values, $E_\text {Na}=53$ mV and $E_\text {Ca}(0)=131.06$ mV. For the altered models, $E_\text {Na}=63$ mV and $E_\text {Ca}(0)=161.53$ mV, $c_\text {m}$ is increased by a factor 1.5 and the conductances are indicated in the legend

As shown in Fig. 6, the experiments of Tewari et al. could be explained through various combinations of changes in a selection of the parameters $c_\text {m}$, ${E}_{\text {Na}}$, ${E}_{\text {Ca}}$ $\bar{g}_{\text {Kv2like}}$ and $\bar{g}_\text {SK}$. Allen model 1 was chosen as that yielded the strongest responses to changes in parameters, and was therefore the most promising candidate for recreating the 38% average drop in *f* from Tewari et al.’s experiments.

In general, achieving a reduction in *f* similar to what was seen in Fig. 1 required quite large changes in several parameters, and a large increase in $c_\text {m}$ was a necessary part of it. In Fig. 6, $c_\text {m}$ was increased by a factor 1.5, $E_{\text {Ca}}$ and $E_{\text {Na}}$ (when included) were shifted by 30 and 10 mV, respectively, while $\bar{g}_{\text {Kv2like}}$ and $\bar{g}_\text {SK}$ were varied (jointly, when both were included) by factors between 1.5 and 4 as indicated in the figure legends. Upregulation of $\bar{g}_\text {SK}$ by such a high factor due to PNN degradation is supported by the experiments by Dembitskaya et al. (2021). It was there found that on average, $\bar{g}_\text {SK}$ increased by a factor three after PNN degradation, but changes up to a factor six was within the standard deviation in the experimental data. For the remaining parameters, the literature gives no guidance as to whether PNN degradation should affect them in the way suggested in Fig. 6.

Not surprisingly, the largest effect on *f* was found when the full set of candidate mechanisms were changed in the same model. When $\bar{g}_{\text {Kv2like}}$ and $\bar{g}_\text {SK}$ were increased by a factor four (relative to their default values in the model), the reduction in *f* exceeded that seen in Fig. 1.

## Discussion

While an increasing number of studies show that degradation of PNNs increases plasticity (Fawcett et al., 2019), the underlying mechanisms remain elusive. An important piece of the puzzle is to reveal the role of PNNs for the neuron’s electrophysiological properties. In the experimental paper by Tewari et al. (2018) it was shown that PNN degradation led to a 25–50% increase in the membrane capacitance $c_\text {m}$, and a decrease in the firing rate *f* of parvalbumin positive (PV) interneurons. In the current study, we showed for a selection of nine computational neuron models, that the reported reduction in $c_\text {m}$ indeed lead to reduced *f*, but could not explain a reduction as large as that seen in the experiments. We therefore hypothesized that the reduction in *f* was due to a combination of cellular mechanisms affected by PNN degradation.

By systematically exploring how *f* was reduced by changes in selected model parameters, we proposed an explanation where the reduced firing rate in Tewari et al.’s experiments is due to (1) the reported change in $c_\text {m}$ combined with (2) upregulation of potassium channels $\bar{g}_{\text {SK}}$ and $\bar{g}_{\text {Kv2like}}$, and (3) upward shifts in Ca$^{2+}$ and Na$^{+}$ reversal potentials. Whereas the upregulation of $\bar{g}_\text {SK}$ is supported by previous experimental data (Dembitskaya et al., 2021), the proposed effects of PNN degradation on the remaining parameters are neither supported nor conflicted by existing literature.

Experimental literature on how PNNs affect ion channels and reversal potentials is sparse. In the few studies that exist, the focus is often on how individual PNN components affect cell properties, and not on PNNs as a an intact structure (see e.g. Xiao et al., (1999), or Favuzzi et al., (2017)). This opens up for speculation about how PNN degradation actually affects the cell. When we in the current study compared our model predictions with experimental findings, we assumed effects of net components were the same when embedded in the net as when applied artificially in a bath solution, and likewise that dissolving PNNs corresponds to degradation of all PNN components. However, we cannot exclude the possibility that components of dissolved nets in reality will be floating around in the extracellular space, having the same (or even stronger) effect on cells as when embedded in the PNNs.

PNNs encapsulate neural membranes inhibiting the growth of new spines (Dansie & Ethell, 2011; Bikbaev et al., 2015). The impact on spine growth provides a quite simple explanation to the relationship between PNNs and $\bar{g}_{\text {SK}}$: PNN degradation would facilitate the growth of new spines, and as $\bar{g}_{\text {SK}}$ are expressed in spines in many neurons, this could lead to a quite dramatic increase $\bar{g}_\text {SK}$ expression (Dembitskaya et al., 2021). The mechanisms through which PNNs should affect the expression or kinetics of ion channels not primarily located in spines are less clear. For simplicity, we assumed that key effects of PNN degradation on ion channels could be modeled as up- or down regulation of conductances (for fully open channels). However, we note that in some cases, net components can have more complex effects on ion channels than mere up- or down-regulation. For instance, net components have been shown to alter the activation curves of Ca$^{2+}$ channels (Vigetti et al., 2008), and in principle, activation time constants could also be affected. Since there is little available data that would allow us to constrain PNN effects on ion channel kinetics, we made the simple choice of only varying the conductances. However, a more in-depth study of effects of PNN degradation on firing rates could be conducted when more data is available on how PNN degradation affects specific mechanisms on the cellular level.

An important feature of Allen model 1 (our selected “main model”) was that the effect on *f* by regulating Ca$^{2+}$ conductances $\bar{g}_\text {HVA}$ and $\bar{g}_\text {LVA}$ was always indirect, i.e. via the activation of $\bar{g}_{\text {SK}}$ by intracellular Ca$^{2+}$. The interplay between Ca$^{2+}$ influx and the activation of Ca$^{2+}$ activated K$^{+}$ channels such as SK is generally intricate (Sah & Faber, 2002; Shin et al., 2022), and we note that the Ca$^{2+}$ conductances in our model could have the opposite effect if $\bar{g}_{\text {SK}}$ were very small or absent in the model. In that case, *f* would decrease with decreasing Ca$^{2+}$ conductances, and not increase, as it did in our simulations. This would reinstate the Ca$^{2+}$ conductances as candidate mechanisms for firing frequency reduction, as a reduction in $I_\text {Ca}$ and *f* has been found upon PNN degradation (Vigetti et al., 2008; Kochlamazashvili et al., 2010). As Allen model 1 instead leads us to suggest $\bar{g}_{\text {SK}}$ as a key candidate mechanism for explaining reduced firing rates in the experiments of Tewari et al. (2018), we would encourage follow-up experiments aimed to verify this finding. Such experiments could for example use immunohistology or patch-clamping combined with SK antagonists or agonists to verify if (i) PNN degradation actually leads to a change in $\bar{g}_{\text {SK}}$ in the relevant neurons, and (ii) whether $\bar{g}_{\text {SK}}$ regulation actually causes pronounced changes in their firing rates.

The idea that PNNs should affect ionic reversal potentials seems plausible since PNNs consist of negatively charged glycans. It has been suggested that these locally immobilized charges can accumulate a reservoir of physiologically relevant cations such as K$^+$, Na$^+$ and Ca$^{2+}$ in the extracellular vicinity of PNN encapsulated neurons (Brückner et al., 1993; Morawski et al., 2015). However, it is not obvious how such a cation reservoir should affect the reversal potentials. One might imagine that the reservoir simply amounts to increased extracellular concentrations of free K$^+$, Na$^+$ and Ca$^{2+}$, which would correspond to depolarized reversal potentials of these ions. Alternatively, one could imagine that the reservoir instead represents a buffering of these ions, hindering them in crossing the membrane, with the possible consequence of more hyperpolarized reversal potentials. So far, we have failed to find experimental evidence for either of the possibilities, and the link between PNN associated glycans and ionic concentrations appears to be anything but trivial. For example, PNN associated glycans have been found to decrease the intracellular Cl$^-$ concentration, and not increase it, as one intuitively might expect based on their negative (extracellular) charge. As a consequence, enzymatic digestion of glycans was found to depolarize the Cl$^-$-reversal potential $E_\text {Cl}$ (Glykys et al., 2014). When it comes to reversal potentials of the other ion species, we have found no clear statements in the literature as to how PNNs should affect them. One might seek some evidence by exploring effects of PNNs on resting membrane potentials, which depend on the weighted reversal potential of all ions that the resting membrane is permeable to. However, PNN degradation has not been consistently found to alter resting membrane potentials in fast-spiking interneurons (Balmer, 2016; Tewari et al., 2018). The lacking impact on resting potentials implies that PNNs either have little impact on ionic reversal potentials, or that they by chance or evolutionary selection affect multiple reversal potentials in concert so that their net effects on the resting membrane is small. In this context, it should be noted that the resting membrane potential is by far most sensitive to $E_\text {K}$ and $E_\text {Cl}$, while $E_\text {Na}$ and especially $E_\text {Ca}$ could in principle change quite a lot without affecting the resting membrane potential much (Hodgkin & Katz, 1949).

We note that PNN degradation is likely to impact many mechanisms besides those considered in the current study. PNNs can for example influence glycan-protein ligand interactions and accessibility to receptors on the neuronal surface (McRae et al., 2012), influence neuron-glia interactions (Carulli et al., 2016), synaptic transmission (Sonntag et al., 2018), and regulate PV expression in itself (Enwright et al., 2016), as might have possible effects on the dynamical properties of the affected neurons. Most of these off-target changes were in the experiments of Tewari et al. (2018) ruled out as the main explanatory effects behind changed firing-rate changes (Tewari et al., 2018), and neither of these effects were considered in the current modeling study.

In general, the parameter changes that affected $f-I$ curves in our models also affected shapes of their action potentials. However, the relationship between the action-potential shapes and $f-I$ curves is generally not trivial. The durations (or widths) of action potentials are in many studies reported to increase with firing frequency (see e.g. Bourque & Renaud (1985) or Stratton et al. (2012)), yet examples of the opposite can also be found (see e.g. Kispersky et al., (2012) or Halnes et al., (2019)). As demonstrated in Supplementary Fig. 4, (panels F–K) for the final set of candidate models (those in Fig. 6), the duration of the action potentials could in our simulations both increase and decrease with firing frequency, depending on position on the stimulus-current axis. However, the overall variations in the action potential-shape were quite moderate, and its relationship to the $f-I$ curves was not studied further here.

Finally, we note that all models are simplifications that are bound to lack some mechanisms present in the real systems. In that context, we note that the $f-I$ curves of fast-spiking interneurons in the Tewari data (Fig. 1) were more linear than the $f-I$ curves of any of our nine considered computational models, both under in control conditions and after PNN-degradation. Hence, none of the models seem to accurately describe the firing properties of the cell type in the experiments, pointing to mechanisms lacking in the models. The ideal starting point for the study presented would thus be the construction of a new multicompartment neuron model, validated against electrophysiological data from the relevant neuron type under the same experimental conditions as in the experiments of Tewari et al. (2018). This would require a large modeling effort including collaboration with experimentalists willing to do the relevant recordings, and was regarded as being beyond the scope of the current study. We instead considered the morphologically detailed state-of-the-art models from the Allen Brain Atlas’ Cell Database as the best candidate models for the neurons in question, as these had passive and active parameters fitted to electrophysiological data from PV neurons in mice, i.e., the same kind of neurons that were targeted in the experiments of Tewari et al. (2018).

## Supplementary Information

Below is the link to the electronic supplementary material.

Supplementary file1 (DOCX 1.81 MB)

## Data Availability

The code used to produce and analyze the results in this paper is archived on Zenodo: https://doi.org/10.5281/zenodo.7688284. It is also available on GitHub: https://github.com/KineOdegardHanssen/NEURON_Allen_capacitance.

## References

[CR1] Abbott, L. F., & Kepler, T. B. (1990). Model neurons: from Hodgkin-Huxley to Hopfield. In *Statistical mechanics of neural networks* (Springer). 5–18

[CR2] Allen Institute for Brain Science. (2017). Technical White Paper: Biophysical Modeling - Perisomatic. http://help.brain-map.org/display/celltypes/Documentation

[CR3] Allen Institute for Brain Science. (2022). Overview: Allen brain atlas: Cell types. http://celltypes.brain-map.org/

[CR4] Balmer, T. S. (2016). Perineuronal nets enhance the excitability of fast-spiking neurons. *eNeuro* 3. 10.1523/ENEURO.0112-16.2016 PMC4987413 27570824

[CR5] Bartos, M. & Elgueta, C. (2012). Functional characteristics of parvalbumin- and cholecystokinin-expressing basket cells. *The Journal of Physiology**590*, 669–681. https://onlinelibrary.wiley.com/doi/pdf/10.1113/jphysiol.2011.226175 10.1113/jphysiol.2011.226175 PMC3381301 22250212

[CR6] Bikbaev A, Frischknecht R, Heine M (2015). Brain extracellular matrix retains connectivity in neuronal networks. Scientific Reports.

[CR7] Bourque C, Renaud L (1985). Activity dependence of action potential duration in rat supraoptic neurosecretory neurones recorded in vitro. The Journal of Physiology.

[CR8] Brückner G, Brauer K, Härtig W, Wolff JR, Rickmann MJ, Derouiche A (1993). Perineuronal nets provide a polyanionic, glia-associated form of microenvironment around certain neurons in many parts of the rat brain. Glia.

[CR9] Burket JA, Webb JD, Deutsch SI (2021). Perineuronal nets and metal cation concentrations in the microenvironments of fast-spiking, parvalbumin-expressing gabaergic interneurons: relevance to neurodevelopment and neurodevelopmental disorders. Biomolecules.

[CR10] Burket, J. A., Urbano, M. R., & Deutsch, S. I. (2022). Sugarcoated perineuronal nets regulate “GABAergic” transmission: Bittersweet hypothesis in autism spectrum disorder. *Clinical Neuropharmacology*, *40*, 120–130. 10.1097/WNF.0000000000000209 28277443

[CR11] Carnevale, N. T. & Hines, M. L. (2006). *The NEURON Book* (Cambridge University Press). 10.1017/CBO9780511541612

[CR12] Carulli, D., Kwok, J. C., & Pizzorusso, T. (2016). [Dataset] Perineuronal nets and cns plasticity and repair. 10.1155/2016/4327082 PMC4771911 26989516

[CR13] Christensen, A. C., Lensjø, K. K., Lepperød, M. E., Dragly, S. -A., Sutterud, H., Blackstad, J. S., et al. (2021). Perineuronal nets stabilize the grid cell network Nature. *Communications,**12*, 253. Number: 1 Publisher: Nature Publishing Group. 10.1038/s41467-020-20241-w 10.1038/s41467-020-20241-w PMC7801665 33431847

[CR14] Dansie LE, Ethell IM (2011). Casting a net on dendritic spines: the extracellular matrix and its receptors. Developmental neurobiology.

[CR15] Dembitskaya Y, Gavrilov N, Kraev I, Doronin M, Tang Y, Li L (2021). Attenuation of the extracellular matrix increases the number of synapses but suppresses synaptic plasticity through upregulation of sk channels. Cell Calcium.

[CR16] Destexhe, A., Mainen, Z. F., & Sejnowski, T. J. (1994). Synthesis of models for excitable membranes, synaptic transmission and neuromodulation using a common kinetic formalism. *Journal of Computational Neuroscience**1*, 195–230. 10.1007/BF00961734 10.1007/BF00961734 8792231

[CR17] Destexhe A, Sejnowski TJ (2003). Interactions between membrane conductances underlying thalamocortical slow-wave oscillations. Physiological reviews.

[CR18] Dityatev, A., Brückner, G., Dityateva, G., Grosche, J., Kleene, R., & Schachner, M. (2007). Activity-dependent formation and functions of chondroitin sulfate-rich extracellular matrix of perineuronal nets *Developmental Neurobiology,**67*, 570–588. https://onlinelibrary.wiley.com/doi/pdf/10.1002/dneu.20361 10.1002/dneu.20361 17443809

[CR19] Enwright JF, Sanapala S, Foglio A, Berry R, Fish KN, Lewis DA (2016). Reduced labeling of parvalbumin neurons and perineuronal nets in the dorsolateral prefrontal cortex of subjects with schizophrenia. Neuropsychopharmacology.

[CR20] Favuzzi E, Marques-Smith A, Deogracias R, Winterflood CM, Sánchez-Aguilera A, Mantoan L (2017). Activity-dependent gating of parvalbumin interneuron function by the perineuronal net protein brevican. Neuron.

[CR21] Fawcett, J. W., Oohashi, T., & Pizzorusso, T. (2019). The roles of perineuronal nets and the perinodal extracellular matrix in neuronal function *Nature Reviews Neuroscience**20*, 451–465. Number: 8 Publisher: Nature Publishing Group. 10.1038/s41583-019-0196-3 10.1038/s41583-019-0196-3 31263252

[CR22] Glykys J, Dzhala V, Egawa K, Balena T, Saponjian Y, Kuchibhotla K (2014). Local impermeant anions establish the neuronal chloride concentration. Science.

[CR23] Hagen, E., Næss, S., Ness, T. V., & Einevoll, G. T. (2018). Multimodal modeling of neural network activity: Computing LFP, ECoG, EEG, and MEG signals with LFPy 2.0 *Frontiers in Neuroinformatics* 12. 10.3389/fninf.2018.00092 PMC6305460 30618697

[CR24] Halnes, G., Augustinaite, S., Heggelund, P., Einevoll, G. T., & Migliore, M. (2011). *A multi-compartment model for interneurons in the dorsal lateral geniculate nucleus PLOS Computational Biology,**7*, e1002160. Publisher: Public Library of Science. 10.1371/journal.pcbi.1002160 10.1371/journal.pcbi.1002160 PMC3182861 21980270

[CR25] Halnes G, Tennøe S, Haug TM, Einevoll GT, Weltzien F-A, Hodne K (2019). A computational model for gonadotropin releasing cells in the teleost fish medaka. PLoS Computational Biology.

[CR26] Hanssen, K. Ø., & Malthe-Sørenssen, A. (2022). Perineuronal nets restrict transport near the neuron surface: A coarse-grained molecular dynamics study. *Frontiers in Computational Neuroscience,**16.* 10.3389/fncom.2022.967735 PMC9714573 36465960

[CR27] Hirono M, Watanabe S, Karube F, Fujiyama F, Kawahara S, Nagao S (2018). Perineuronal nets in the deep cerebellar nuclei regulate GABAergic transmission and delay eyeblink conditioning. The Journal of Neuroscience.

[CR28] Hodgkin AL, Katz B (1949). The effect of sodium ions on the electrical activity of the giant axon of the squid. The Journal of physiology.

[CR29] Kager H, Wadman WJ, Somjen GG (2000). Simulated seizures and spreading depression in a neuron model incorporating interstitial space and ion concentrations. Journal of Neurophysiology.

[CR30] Kispersky TJ, Caplan JS, Marder E (2012). Increase in sodium conductance decreases firing rate and gain in model neurons. Journal of Neuroscience.

[CR31] Kochlamazashvili, G., Henneberger, C., Bukalo, O., Dvoretskova, E., Senkov, O., Lievens, P. M. J., et al. (2010). The extracellular matrix molecule hyaluronic acid regulates hippocampal synaptic plasticity by modulating postsynaptic l-type ca2+ channels. *Neuron* 67, 116–128. 10.1016/j.neuron.2010.05.030 PMC3378029 20624596

[CR32] Lensjø KK, Lepperød ME, Dick G, Hafting T, Fyhn M (2017). Removal of perineuronal nets unlocks juvenile plasticity through network mechanisms of decreased inhibition and increased gamma activity. The Journal of Neuroscience.

[CR33] McRae PA, Baranov E, Rogers SL, Porter BE (2012). Persistent decrease in multiple components of the perineuronal net following status epilepticus. European Journal of Neuroscience.

[CR34] Morawski M, Reinert T, Meyer-Klaucke W, Wagner FE, Tröger W, Reinert A (2015). Ion exchanger in the brain: Quantitative analysis of perineuronally fixed anionic binding sites suggests diffusion barriers with ion sorting properties Scientific Reports.

[CR35] Pizzorusso, T., Medini, P., Berardi, N., Chierzi, S., Fawcett, J. W., & Maffei, L. (2002). Reactivation of ocular dominance plasticity in the adult visual cortex. *Science,**298*. 10.1126/science.1072699. 10.1126/science.1072699 12424383

[CR36] Pyka, M., Wetzel, C., Aguado, A., Geissler, M., Hatt, H., & Faissner, A. (2011). Chondroitin sulfate proteoglycans regulate astrocyte-dependent synaptogenesis and modulate synaptic activity in primary embryonic hippocampal neurons *European Journal of Neuroscience**33*, 2187–2202. https://onlinelibrary.wiley.com/doi/pdf/10.1111/j.1460-9568.2011.07690.x 10.1111/j.1460-9568.2011.07690.x 21615557

[CR37] Sætra MJ, Einevoll GT, Halnes G (2020). An electrodiffusive, ion conserving Pinsky-Rinzel model with homeostatic mechanisms. PLOS Computational Biology.

[CR38] Shin, J., Kovacheva, L., Thomas, D., Stojanovic, S., Knowlton, C. J., Mankel, J., et al. (2022). Cav1.3 calcium channels are full-range linear amplifiers of firing frequencies in lateral DA SN neurons. *Science Advances,* *8*, eabm4560 10.1126/sciadv.abm4560 PMC9177074 35675413

[CR39] Sorg BA, Berretta S, Blacktop JM, Fawcett JW, Kitagawa H, Kwok JC (2016). Casting a wide net: Role of perineuronal nets in neural plasticity. The Journal of Neuroscience.

[CR40] Srinivasan, J., Schachner, M., & Catterall, W. A. (1998). Interaction of voltage-gated sodium channels with the extracellular matrix molecules tenascin-C and tenascin-R *Proceedings of the National Academy of Sciences* 95, 15753–15757. Publisher: Proceedings of the National Academy of Sciences. 10.1073/pnas.95.26.15753. 10.1073/pnas.95.26.15753 PMC28116 9861042

[CR41] Sterratt, D., Graham, B., Gillies, A., & Willshaw, D. (2011). *Principles of Computational Modelling in Neuroscience* (Cambridge University Press), 1. edn.

[CR42] Szlavik RB (2003). Strategies for improving neural signal detection using a neural-electronic interface. IEEE Transactions on neural systems and rehabilitation engineering.

[CR43] Sah P, Faber EL (2002). Channels underlying neuronal calcium-activated potassium currents. Progress in neurobiology.

[CR44] Sonntag M, Blosa M, Schmidt S, Reimann K, Blum K, Eckrich T (2018). Synaptic coupling of inner ear sensory cells is controlled by brevican-based extracellular matrix baskets resembling perineuronal nets. BMC Biology.

[CR45] Stratton P, Cheung A, Wiles J, Kiyatkin E, Sah P, Windels F (2012). Action potential waveform variability limits multi-unit separation in freely behaving rats. PloS One.

[CR46] Tewari, B. P., Chaunsali, L., Campbell, S. L., Patel, D. C., Goode, A. E., & Sontheimer H. (2018). Perineuronal nets decrease membrane capacitance of peritumoral fast spiking interneurons in a model of epilepsy. *Abstract Nature Communications*, *9*(1). 10.1038/s41467-018-07113-0 10.1038/s41467-018-07113-0 PMC6226462 30413686

[CR47] van ’t Spijker, H. M. & Kwok, J. C. F. (2017). A sweet talk: The molecular systems of perineuronal nets in controlling neuronal communication *Frontiers in Integrative Neuroscience* 11. 10.3389/fnint.2017.00033 PMC5717013 29249944

[CR48] Xiao, Z. -C., Ragsdale, D. S., Malhotra, J. D., Mattei, L. N., Braun, P. E., Schachner, M., et al. (1999). Tenascin-R is a functional modulator of sodium channel $$\beta$$ subunits. *Journal of Biological Chemistry* 274, 26511–26517. 10.1074/jbc.274.37.26511 10473612

[CR49] Vigetti, D., Andrini, O., Clerici, M., Negrini, D., Passi, A., & Moriondo, A. (2008). Chondroitin sulfates act as extracellular gating modifiers on voltage-dependent ion channels. *Cellular Physiology and Biochemistry,**22*, 137–146. Publisher: Karger Publishers. 10.1159/000149791 10.1159/000149791 18769040

[CR50] Wang L, Liu S, Zhang J, Zeng Y (2012). Burst firing transitions in two-compartment pyramidal neuron induced by the perturbation of membrane capacitance. Neurological Sciences.

[CR51] Wei Y, Ullah G, Schiff SJ (2014). Unification of neuronal spikes, seizures, and spreading depression. Journal of Neuroscience.

