# Supplementary material for: Responses in fast-spiking interneuron firing rates to parameter variations associated with degradation of perineuronal nets
Source: J Comput Neurosci. 2023 Apr 14;51(2):283–98. doi: 10.1007/s10827-023-00849-9 (PMC10182141; doi:10.1007/s10827-023-00849-9)
Supplement: Supplementary file 1 — Supplementary file1 (DOCX 1.81 MB) [file 10827_2023_849_MOESM1_ESM.docx]

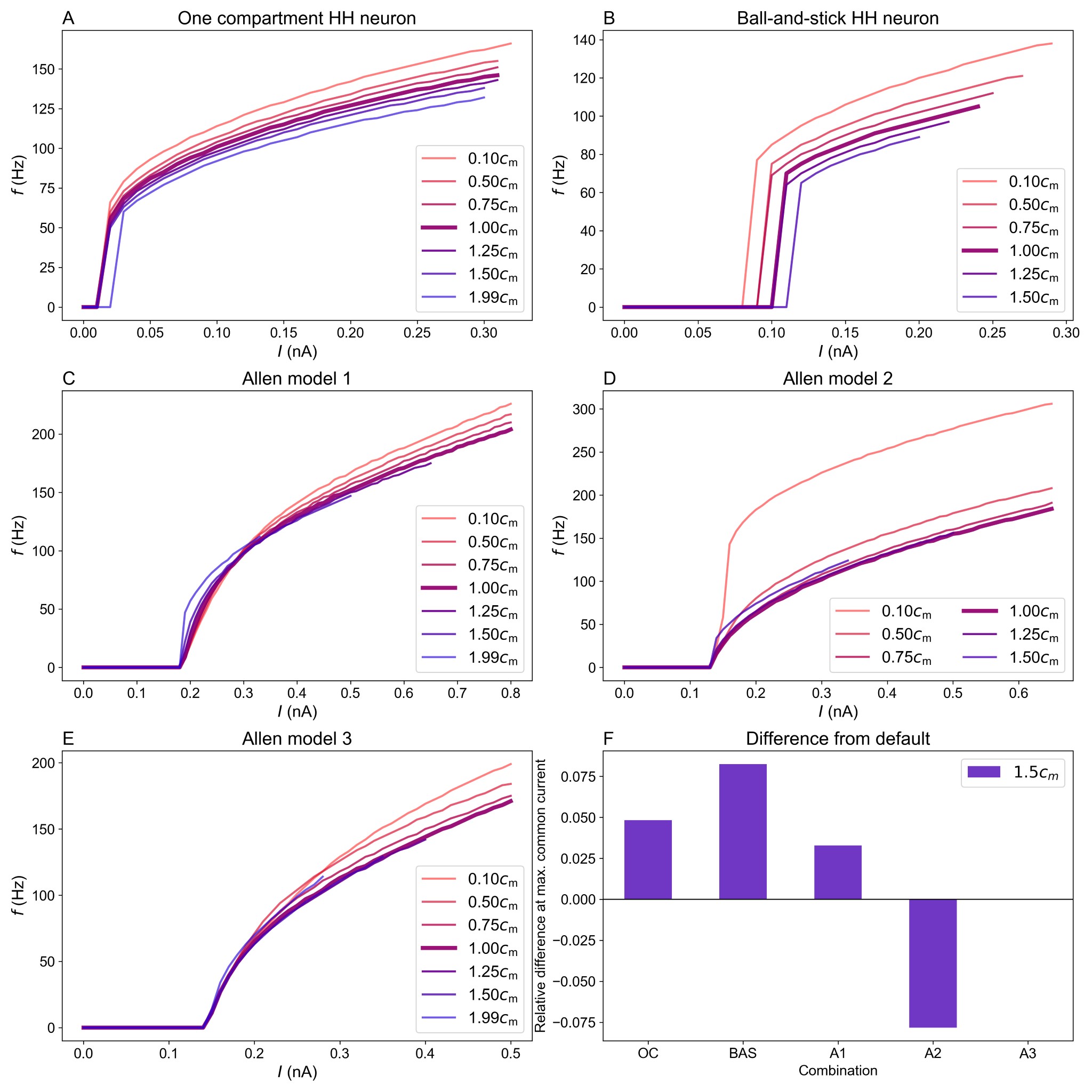


Supplementary Figure 1: Frequency-input curves for selected values of *c*_m_ for the various models. *c*_m_ is altered everywhere. A) The one-compartment Hodgkin-Huxley model, B) The ball-and-stick Hodgkin-Huxley model,

C) Allen model 1, D) Allen model 2, E) Allen model 3, F) The relative difference in *f* between the 1*.*0*c*_m_- and 1*.*5*c*_m_ curves computed at the largest current that gave sustained firing in both cases


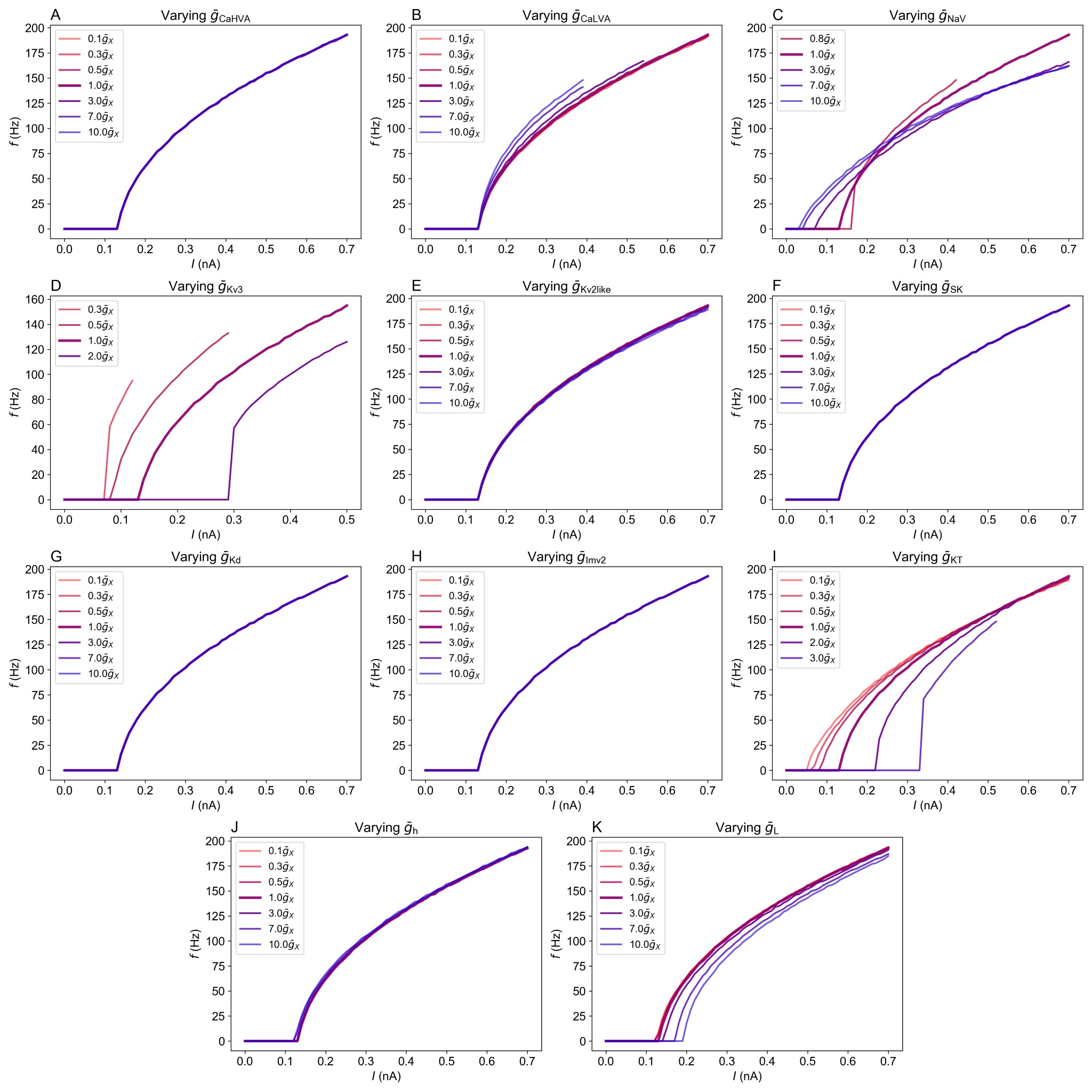


Supplementary Figure 2: Frequency-input curves when varying different conductances in Allen model 2. ¯*g_X_* is the default value of the conductance. A) ¯*g*_CaHVA_, B) ¯*g*_CaLVA_, C) ¯*g*_NaV_, D) ¯*g*_Kv3_, E) ¯*g*_Kv2like_, F) ¯*g*_SK_, G) ¯*g*_Kd_, H) *g*¯_Imv2_, I) ¯*g*_KT_, J) ¯*g*_h_, K) ¯*g*_L_

1


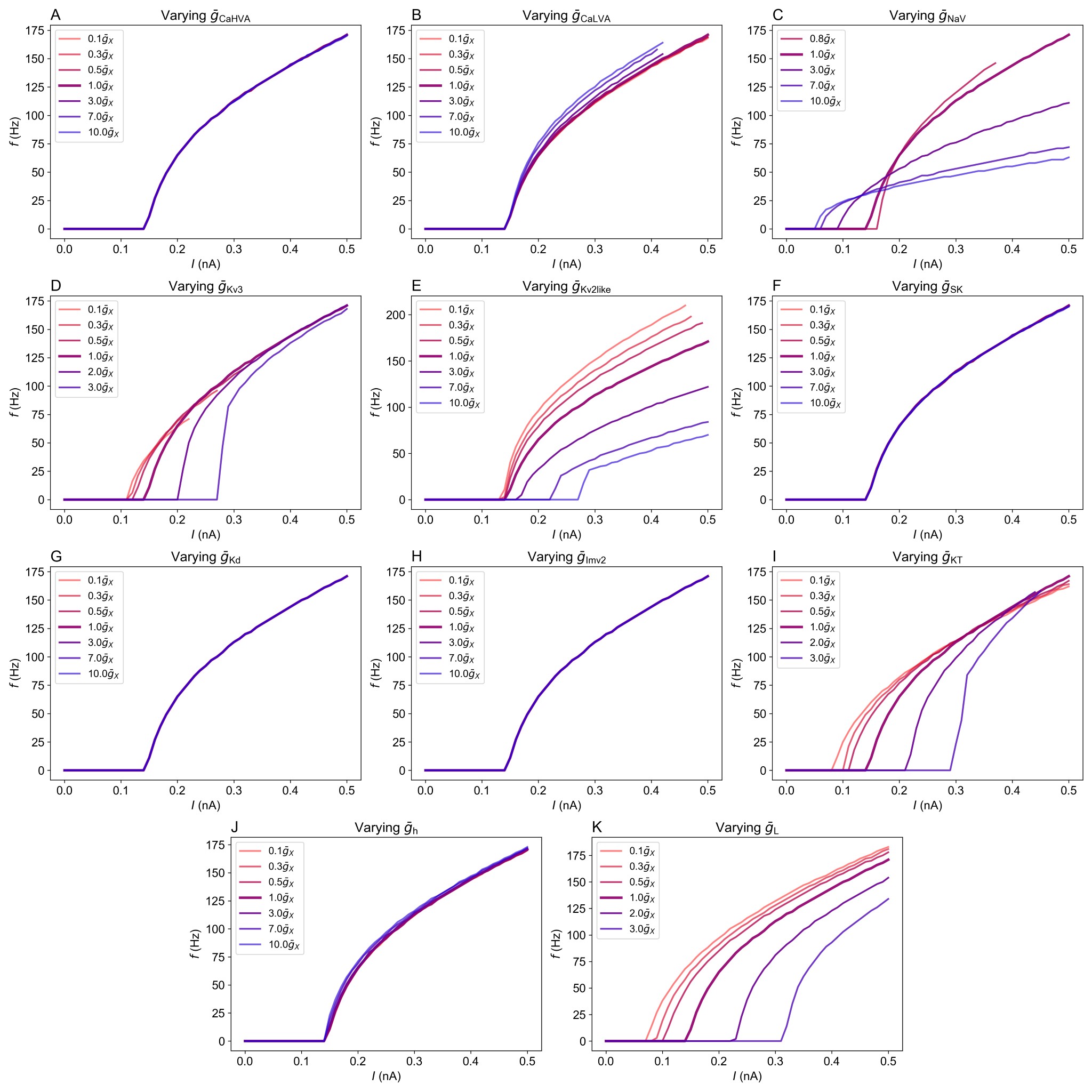


Supplementary Figure 3: Frequency-input curves when varying different conductances in Allen model 3. ¯*g_X_* is the default value of the conductance. A) ¯*g*_CaHVA_, B) ¯*g*_CaLVA_, C) ¯*g*_NaV_, D) ¯*g*_Kv3_, E) ¯*g*_Kv2like_, F) ¯*g*_SK_, G) ¯*g*_Kd_, H) *g*¯_Imv2_, I) ¯*g*_KT_, J) ¯*g*_h_, K) ¯*g*_L_

1


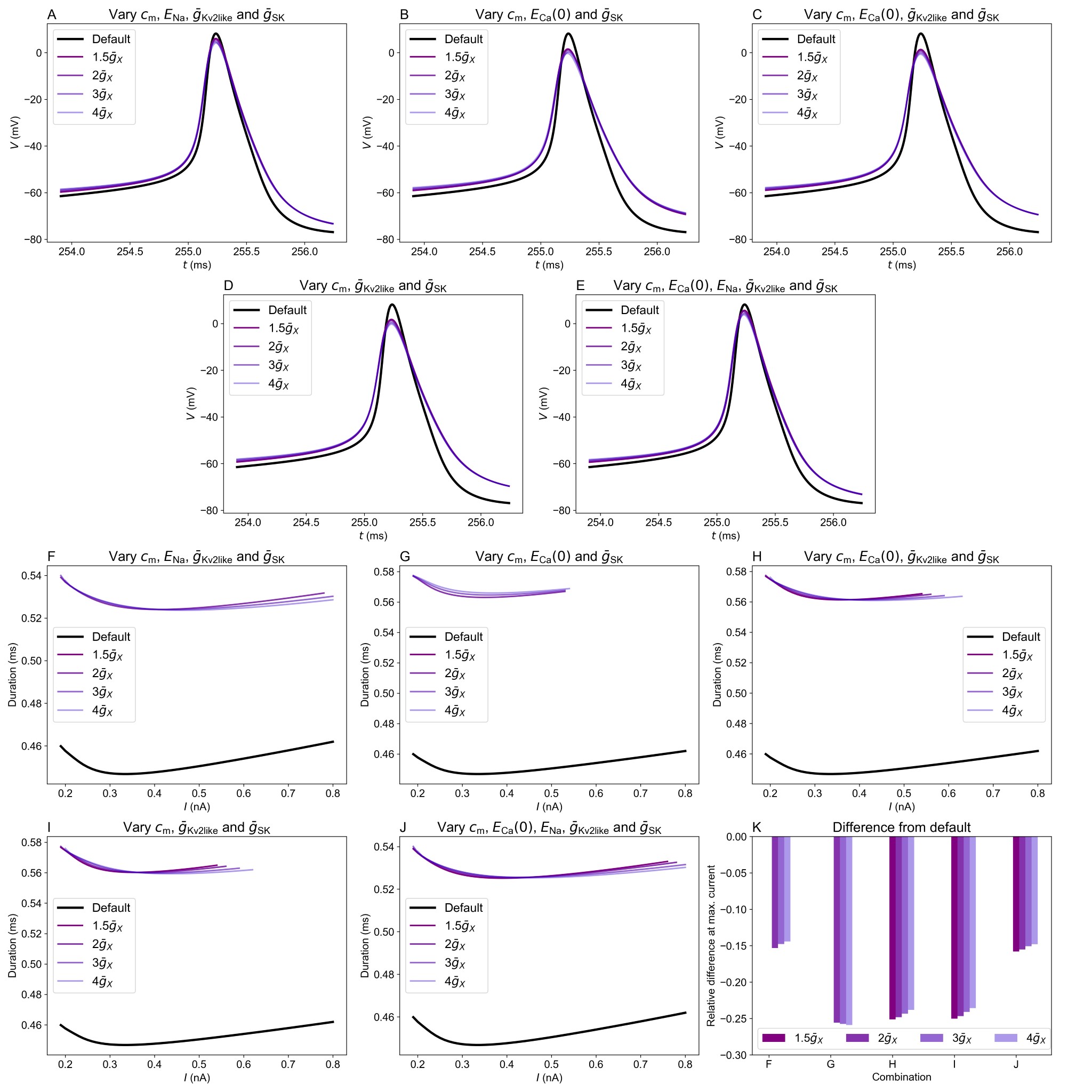


Supplementary Figure 4: Changes in spike shape in Allen model 1 when varying the parameter sets of Figure 6. ¯*g_X_* is the default value of the conductances. A)-E) The membrane potential over one spike at *I* = 0.5 nA. The spikes have been shifted in order to overlap, F-J) Spike duration at -40 mV vs *I*, K) Difference in spike duration between each parameter combination and default, at the highest input current where both are firing. The parameter combinations in each panel are *c*_m_ and A),F) *E*_Na_, ¯*g*_Kv2like_ and ¯*g*_SK_, B),G) *E*_Ca_(*t* = 0) and ¯*g*SK C),H) *E*Ca(*t* = 0), ¯*g*Kv2like and ¯*g*SK D),I) ¯*g*Kv2like and ¯*g*SK E),J) *E*Ca(*t* = 0), *E*Na, ¯*g*Kv2like and ¯*g*SK. Default - default values, *E*_Na_ = 53 mV and *E*_Ca_(0) = 131*.*06 mV. For the altered models, *E*_Na_ = 63 mV and *E*_Ca_(0) = 161*.*53 mV, *c*_m_ is increased by a factor 1.5 and the conductances are indicated in the legend

1

1
